# The genus *Trachionus* Haliday, 1833 (Hymenoptera, Braconidae, Alysiinae) new for China, with description of four new species

**DOI:** 10.3897/zookeys.512.9759

**Published:** 2015-07-06

**Authors:** Qian Cui, Cornelis van Achterberg, Jiang-Li Tan, Xue-Xin Chen

**Affiliations:** 1 Key Laboratory of Resource Biology and Biotechnology in Western China (Northwest University), Ministry of Education; College of Life Sciences, Northwest University, 229 North Taibai Road, Xi’an, Shaanxi 710069, China; 2 Institute of Insect Sciences, Zhejiang University, Zijingang Campus, Yuhangtang Road 866, Hangzhou 310058, China

**Keywords:** Braconidae, Alysiinae, Dacnusini, *Trachionus*, new species, China, Shaanxi

## Abstract

The genus *Trachionus* Haliday, 1833 (Hymenoptera, Braconidae, Alysiinae, Dacnusini) is reported for the first time from China. The genus is represented by four new species from Shaanxi province (NW China), which are described and illustrated. An identification key to the species in China is presented, a key to the genera of the *Trachionus* group and notes on the relationships with other Palaearctic species are added.

## Introduction

*Trachionus* Haliday, 1833 (Hymenoptera, Braconidae, Alysiinae, Dacnusini) is a small Holarctic genus with seven Palaearctic species (four of which reported from the East Palaearctic region) and six Nearctic species. Species of *Trachionus* are parasitoids of the larvae of the genus *Phytobia* Lioy, 1864 (Diptera: Agromyzidae) mining in or near the cambium of trees and shrubs (Yu et al. 2012; for a review, see van Achterberg et al. 2012). The most recent key to the Palaearctic species is by Perepechayenko (2000) and a recent key to the Nearctic species is lacking. In the present paper, we describe four species of this genus collected during fieldwork by one of us (JLT); which proved all to be new to science. It is the first report of the genus from China, but the genus is known from neighbouring countries: Russia (including Far East region), Mongolia, Japan and Korea (Yu et al. 2012).

## Material and methods

The specimens were collected by hand net during fieldwork in the Qinling Mountains in Shaanxi province (Northwest China). The specimens were collected directly in alcohol and later prepared with the AXA method (van Achterberg 2009: method applying first a mixture of alcohol 96% + xylene, after 24 hours replaced by amylacetate).

Morphological terminology follows van Achterberg (1988, 1993), including the abbreviations for the wing venation. Measurements are taken as indicated by van Achterberg (1988): for the length and the width of a body part the maximum length and width is taken, unless otherwise indicated. The length of the mesosoma is measured from the anterior border of the mesoscutum till the apex of the propodeum and of the first tergite from the posterior border of the adductor till the medio-posterior margin of the tergite.

The specimens are deposited in the following collections: Northwest University (NWUX), Xi’an; Institute of Insect Sciences, Zhejiang University (ZJUH), Hangzhou; and Naturalis Biodiversity Center (RMNH), Leiden.

## Descriptions

### Trachionus

Taxon classificationAnimaliaHymenopteraBraconidae

Haliday, 1833

Figs 1 , 2–10 , 11 , 12–21 , 22 , 23–32 , 33 , 34–42 , 43–46

Trachionus Haliday, 1833: 265; Dalla Torre 1898: 198 (as synonym of *Chelonus* Panzer, 1806; *Chelonus mandibularis* attributed to Haliday, and this undescribed species was synonymized (p. 204) with *Chelonus maculator* (Dahlbom, 1833)); Shenefelt 1973: 839 (id.); Marsh 1979a: 231 (id.); van Achterberg 1997: 81 (as valid genus). Type species (by original designation): “*Chelonus mandibularis*” (= *Sigalphus mandibularis* Nees, 1816).
Aenone Haliday, 1833: 267 (nom. nud.; not *Aenone* Lamarck, 1818), 1838: 214; Shenefelt 1974: 1109; van Achterberg 1997: 81 (synonymy with *Trachionus* Haliday).
Aenone Curtis, 1837: 123 (not *Aenone* Lamarck, 1818); van Achterberg 1997: 81 (synonymy with *Trachionus* Haliday). Type species (by present designation): *Sigalphus mandibularis* Nees, 1816.
Oenone Haliday, 1839: 3 (not *Oenone* Lamarck, 1818); Shenefelt 1974: 1109; van Achterberg 1997: 81 (synonymy with *Trachionus* Haliday). Type species (designated by Haliday 1840): *Sigalphus mandibularis* Nees, 1816.
Symphya Foerster, 1863: 273; Shenefelt 1974: 1109–1111; Marsh 1979: 217; van Achterberg 1997: 81 (synonymy with *Trachionus* Haliday). Type species (by original designation): *Sigalphus mandibularis* Nees, 1816.
Anarmus Ruthe (in Brischke), 1882: 138; Shenefelt 1974: 1109; van Achterberg 1997: 81 (synonymy with *Trachionus* Haliday). Type species (by van Achterberg 1997): *Sigalphus mandibularis* Nees, 1816.
Planiricus Perepechayenko, 2000: 30 (as subgenus of *Trachionus*). Type species (by original designation): *Sigalphus hians* Nees, 1816.

#### Diagnosis.

**Figure 1.**
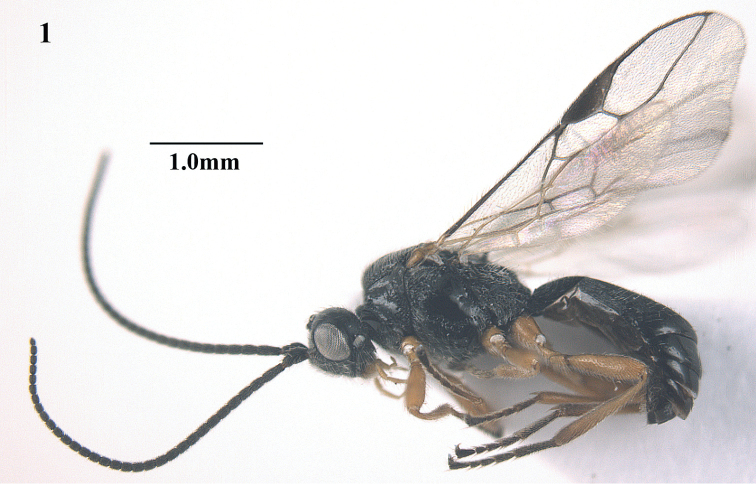
*Trachionus acarinatus* sp. n., male, holotype, habitus lateral.

**Figures 2–10.**
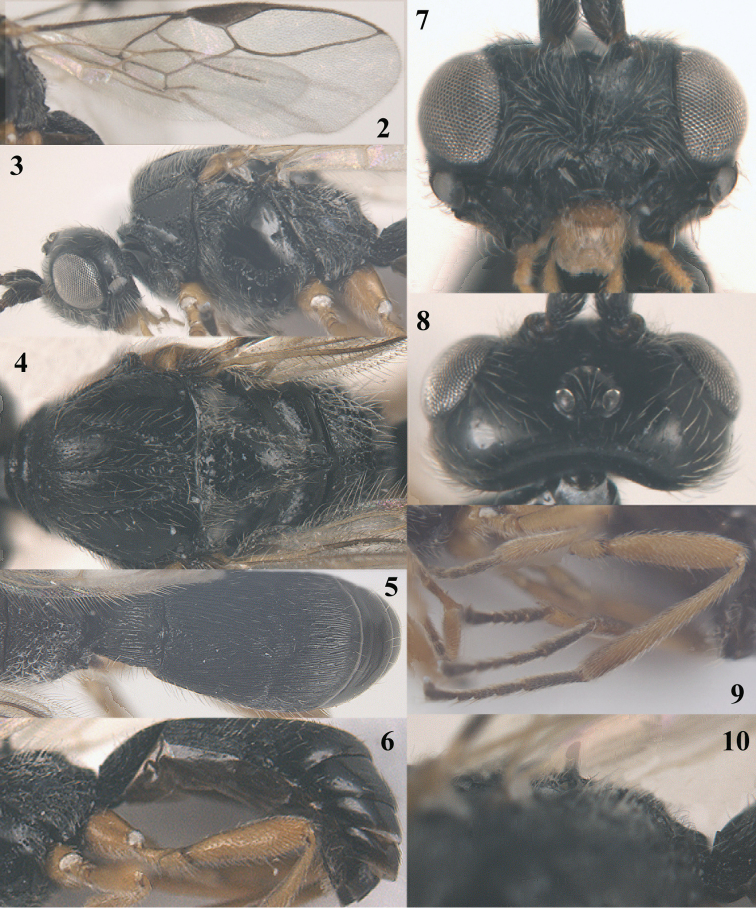
*Trachionus acarinatus* sp. n., male, holotype. **2** wings **3** head and mesosoma lateral **4** mesosoma dorsal **5** metasoma dorsal **6** metasoma lateral **7** head anterior **8** head dorsal **9** hind leg **10** detail of metanotal spine.

**Figure 11.**
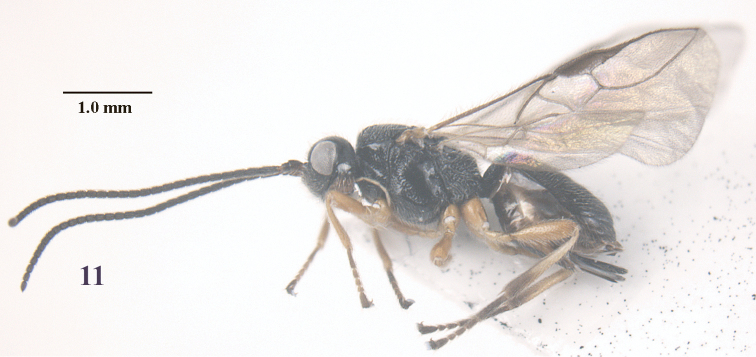
*Trachionus albitibialis* sp. n., female, holotype, habitus lateral.

**Figures 12–21.**
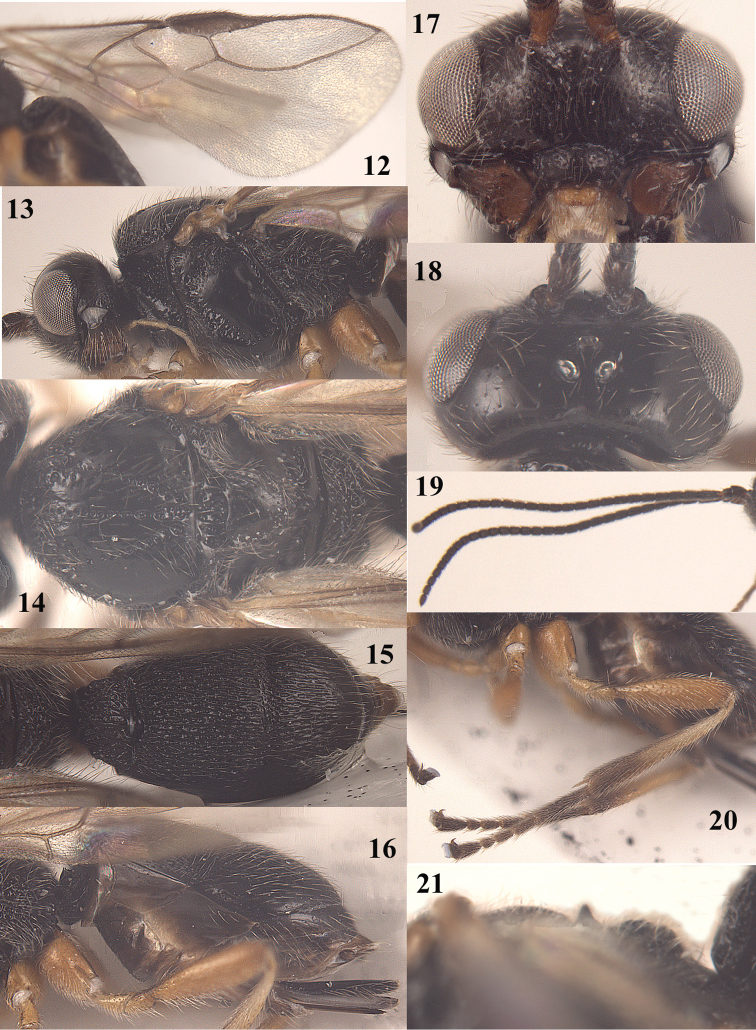
*Trachionus albitibialis* sp. n., female, holotype. **12** wings **13** head and mesosoma lateral **14** mesosoma dorsal **15** metasoma dorsal **16** metasoma lateral **17** head anterior **18** head dorsal **19** antenna **20** hind leg **21** detail of metanotal spine.

**Figure 22.**
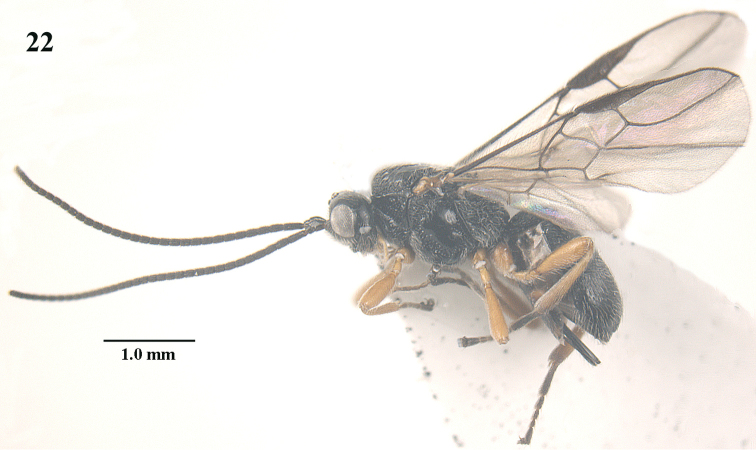
*Trachionus brevisulcatus* sp. n., female, holotype, habitus lateral.

**Figures 23–32.**
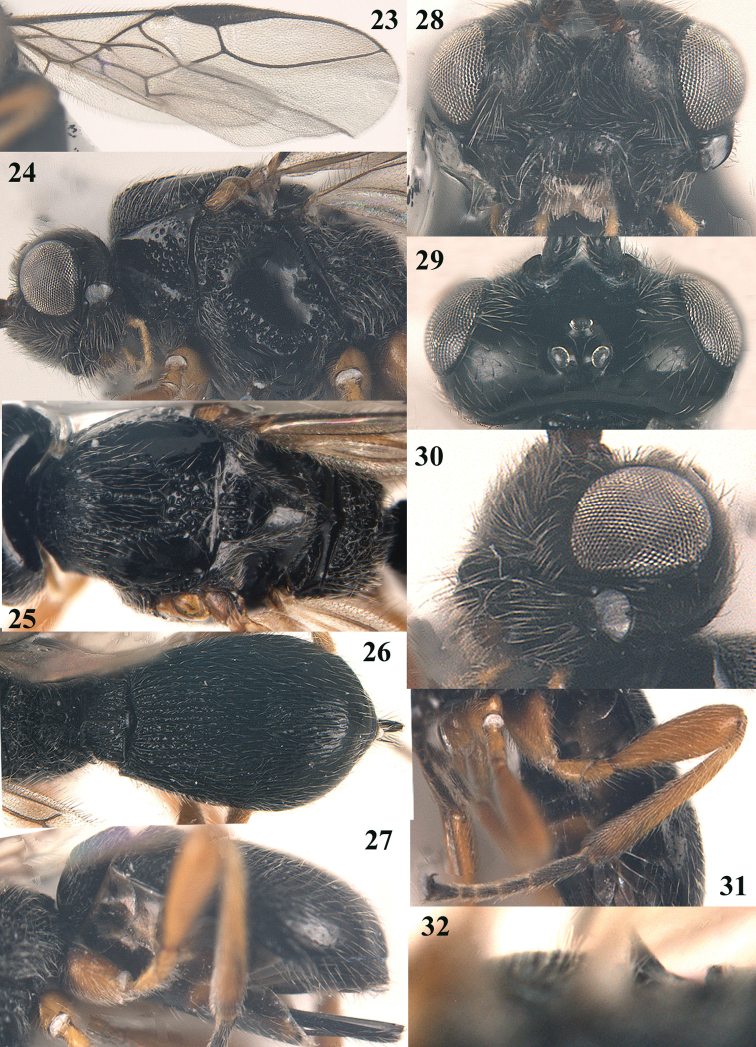
*Trachionus brevisulcatus* sp. n., female, holotype. **23** wings **24** head and mesosoma lateral **25** mesosoma dorsal **26** metasoma dorsal **27** metasoma lateral **28** head anterior **29** head dorsal **30** head lateral **31** hind leg **32** detail of metanotal spine.

**Figure 33.**
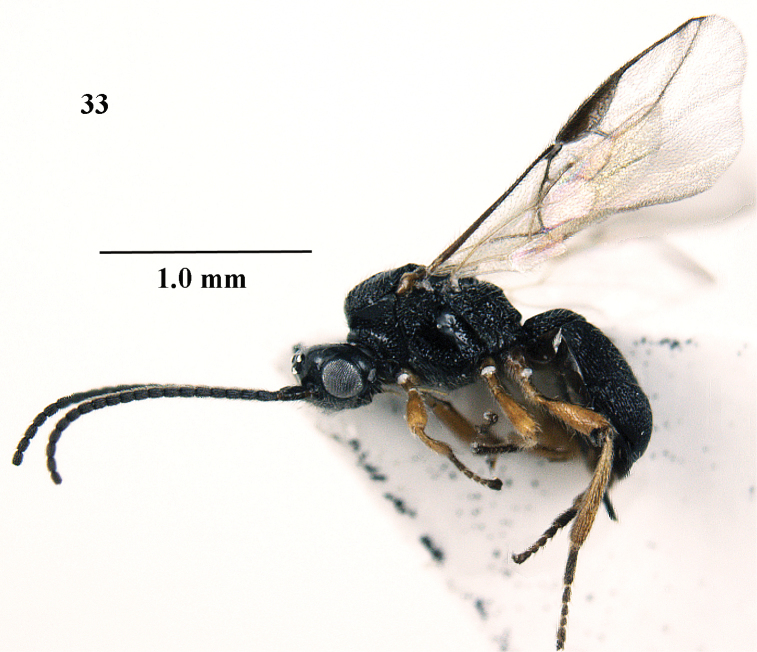
*Trachionus mandibularoides* sp. n., female, holotype, habitus lateral.

**Figures 34–42.**
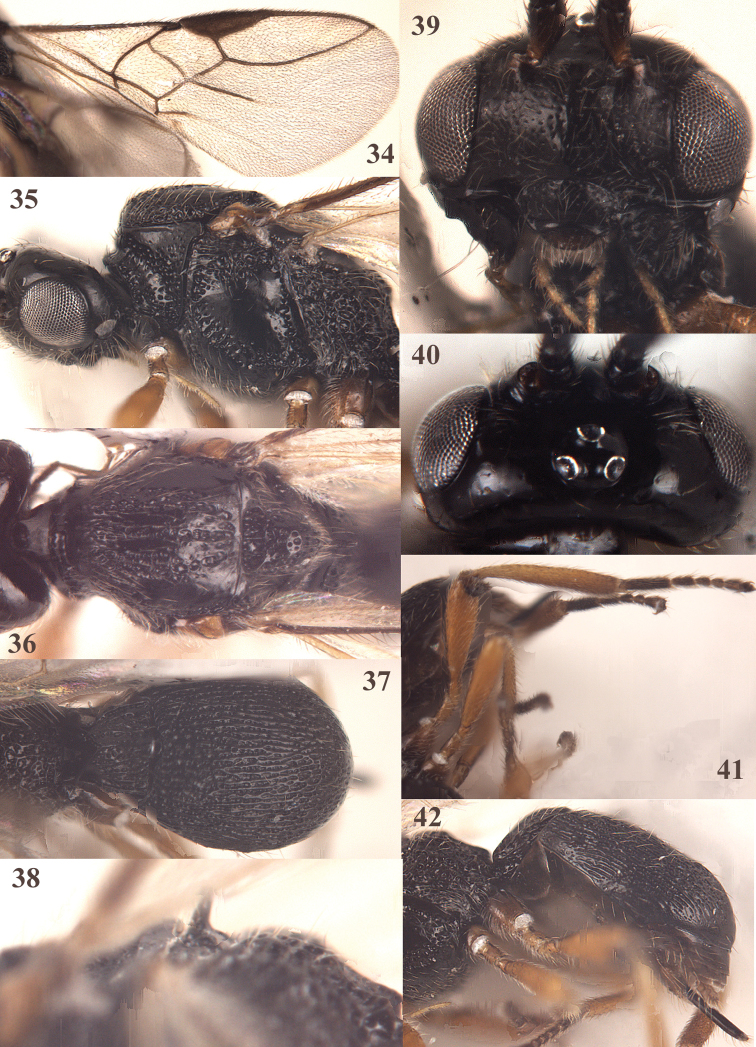
*Trachionus mandibularoides* sp. n., female, holotype. **34** wings **35** head and mesosoma lateral **36** mesosoma dorsal **37** metasoma dorsal **38** detail of metanotal spine **39** head anterior **40** head dorsal **41** hind leg **42** metasoma lateral.

Vein r-m of forewing absent; mandible exodont, with 3–5 teeth or small lobes and second tooth of mandible acute (Figs 7, 17, 28, 30, 39); forewing vein 1-SR longer than 3× width of vein and distinctly longer than parastigma (Figs 1–2, 11–12, 22–23, 33–34); clypeus transverse (Figs 7, 17, 28, 39) and epistomal suture narrow (subgenus *Planiricus* Perepechayenko) or as wide as convex part of clypeus and deep (subgenus *Trachionus* Haliday); ocelli normal (subgenus *Planiricus* Perepechayenko) or strongly protruding (subgenus *Trachionus* Haliday); pronope absent; metanotum distinctly and acutely protruding dorsally (Figs 4, 14, 25, 36); anterior half of sternaulus (below the precoxal sulcus) present or absent, only in subgenus *Trachionus* Haliday posterior half of sternaulus wide and coarsely crenulate; combined length of second and third metasomal tergites of female 0.6–0.8× total length of metasoma, these tergites at least partly sculptured (Figs 5–6, 15–16, 26–27, 37, 42); fourth and fifth metasomal tergites smooth and of female more or less retracted (Figs 16, 27, 42).

#### Biology.

Ovo-larval koinobiont parasitoids of *Phytobia* spp. (Agromyzidae) boring in or near the cambium layer in branches of shrubs and trees (Greene 1914; Clausen 1954; van Achterberg et al. 2012).

#### Notes.

Up to 1997 the interpretation of the genus *Trachionus* had been problematical, not the least because of the synonymy with the genus *Chelonus* by Dalla Torre (1898). It is contradicted by the clear original diagnosis by Haliday (1833): “*Areolae cubitales 2; mandibulae hiantes 4-dentes*”. If the diagnosis of the genus is combined with the diagnosis of the group (“*Abdominis segmenta coalita postica retracta.* Chelonus”) then it is clear that it can only apply to the tribe Dacnusini (because of the exodont mandibles) and to the genus *Aenone* Curtis, 1837 (because of the Chelonine-like metasoma), a junior homonym and, therefore, unavailable. In 1997 van Achterberg clarified the position of the genus *Trachionus* and synonymized *Trachionus* with *Symphya* Foerster, the oldest available name for the group in current use that time. All four species from China belong to the subgenus *Planiricus* Perepechayenko (= *Trachionus hians*-group) because they have the epistomal suture much narrower than the clypeus and the sternaulus is absent or is only anteriorly shallowly developed as punctate area.

*Trachionus* is similar to two Palaearctic genera, *Epimicta* Foerster, 1863, and *Parasymphya* Tobias, 1998, because of the presence of the pronotal spine, and the sculpture and shape of the second and third metasomal tergites. These taxa can be separated as follows:

**Table:** 

1	Clypeus semi-circular; fourth and fifth tergites of ♀ sculptured and more or less exposed; second tooth of mandible obtuse, lobe-shaped; stemmaticum depressed	***Parasymphya* Tobias, 1998**
	Type species and only known species: *Parasymphya dentata* Tobias, 1998. The biology of this East Palaearctic genus is unknown.	
–	Clypeus transverse (Figs 7, 17, 28, 39); fourth and fifth metasomal tergites of ♀ smooth and more or less retracted (Figs 6, 16, 27, 42); second tooth of mandible acute (Figs 7, 17, 28, 30, 39); stemmaticum normal (Figs 8, 18, 29, 40); [parasitoids of Agromyzidae (*Phytobia* spp.) boring in cambium of trees and shrubs]	**2**
2	Pronotal side without strong oblique carina (Figs 3, 13, 24, 35); combined length of second and third metasomal tergites of ♀ 0.6–0.8× total length of metasoma (Figs 5–6, 15–16, 26–27, 37, 42); fourth tergite of ♀ largely or partly retracted and distinctly shorter than second tergite (Figs 1, 5–6, 11, 15–16, 22, 26–27); metanotum distinctly and acutely protruding dorsally (Figs 4, 14, 25, 36); clypeus narrower and distinctly convex (Figs 7, 17, 28, 39); vein 1-SR of forewing long (Figs 1–2, 11–12, 22–23, 33–34)	***Trachionus* Haliday, 1833**
–	Pronotal side with strong oblique carina; combined length of second and third tergites of ♀ 0.3–0.5× total length of metasoma; fourth tergite of ♀ entirely exposed and about as long as second tergite; metanotum at most slightly protruding dorsally; clypeus wide and rather flat; vein 1-SR of forewing medium-sized to short	***Epimicta* Foerster, 1863**

#### Key to Chinese species of *Trachionus* Haliday

**Table:** 

1	Scutellum coarsely punctate and distinctly convex (Fig. 36); sternaulus indicated as punctate area (below precoxal sulcus anteriorly; Fig. 35); median punctate band of mesoscutum anteriorly as wide as smooth bands next to it (Fig. 36)	***Trachionus mandibularoides* sp. n.**
–	Scutellum largely smooth, at most punctulate and nearly flat (Fig. 4); sternaulus absent (Fig. 24); median punctate band of mesoscutum anteriorly narrower than smooth bands next to it (Figs 4, 14, 25)	**2**
2	Apical half of second metasomal tergite regularly and rather finely striate, with about 60 striae and moderately shiny (Fig. 5); metanotal spine long, its highest point reaching level of tips of setae of scutellum (Fig. 10); propodeum gradually lowered posteriorly in lateral view and carina distinctly protruding postero-laterally (Fig. 4); mandible without fourth ventral tooth (Fig. 46)	***Trachionus acarinatus* sp. n.**
–	Apical half of second tergite coarsely rugose-striate, with about 30 striae and very shiny (Fig. 26); metanotal spine medium-sized, its highest point remaining below level of tips of setae of scutellum (Fig. 32); propodeum angularly lowered posteriorly in lateral view and carina hardly protruding postero-laterally (Fig. 25); mandible with fourth ventral tooth or lobe (Figs 43, 45)	**3**
3	Mandible black, medially with irregular transverse crest and with minute fourth and fifth teeth (Figs 28, 30, 45); medial third of hind tibia brownish yellow (Fig. 31); propleuron without transverse carina subposteriorly (Fig. 24); transverse carina of propodeum coarsely developed and irregular; notauli wide posteriorly (Fig. 25)	***Trachionus brevisulcatus* sp. n.**
–	Mandible mainly brown, flat, medially without crest and with medium-sized fourth tooth (Figs 17, 43); medial third of hind tibia ivory (Fig. 20); propleuron with transverse carina subposteriorly (Fig. 13); transverse carina of propodeum indistinct or absent (Fig. 14); notauli rather narrow posteriorly (Fig. 14)	***Trachionus albitibialis* sp. n.**

**Figure 43–46.**
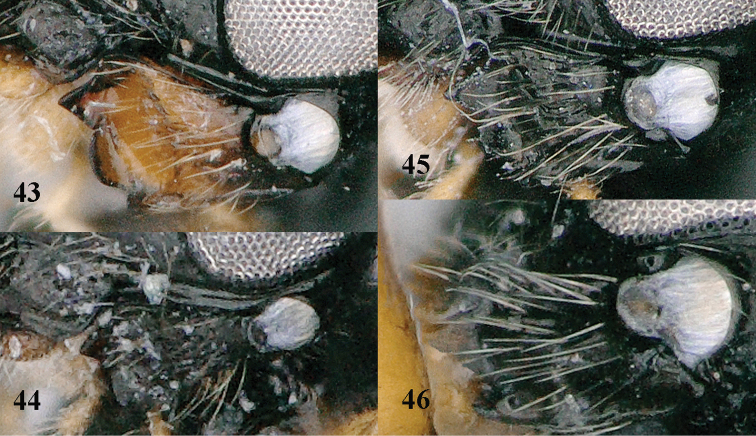
**43** *Trachionus albitibialis* sp. n., female, holotype **44** *Trachionus mandibularoides* sp. n., female, holotype **45** *Trachionus brevisulcatus* sp. n., female, holotyp **46** *Trachionus acarinatus* sp. n., male, holotype **43–46** mandible lateral.

### Trachionus acarinatus

Taxon classificationAnimaliaHymenopteraBraconidae

Cui & van Achterberg sp. n.

http://zoobank.org/350F6D5C-53B4-44EA-A708-6BBD56A0581C

Figs 1 , 2–10 , 46

#### Type material.

Holotype, ♂ (NWUX), “NW. **China**: Shaanxi, Pingheliang, Ningshan, c. 2000 m, 27.vi.2011, 33°48'N 108°50'E, Jiangli Tan, NWUX”. Paratype: 1 ♂ (ZJUH), topotypic and same data.

#### Diagnosis.

Scutellum largely smooth and nearly flat; mandible without fourth ventral tooth (Fig. 46); metanotal spine long, its highest point reaching level of tips of setae of scutellum (Fig. 10); propleuron without distinct transverse carina subposteriorly; precoxal sulcus and notauli medium-sized; propodeum gradually lowered posteriorly in lateral view and carina distinctly protruding postero-laterally (Fig. 4); second and third tergites regularly and rather finely striate, with about 60 striae and moderately shiny (Fig. 5). Similar to *Trachionus hians* (Nees, 1816), but that species has a strong transverse carina at the propleuron subposteriorly (absent or slightly developed in *Trachionus acarinatus*); precoxal sulcus and posterior part of notauli wide (medium-sized) and metanotal spine hardly protruding above level of scutellum (protruding far above level of scutellum).

#### Description.

Holotype, male; body length 3.4 mm, forewing length 3.3 mm.

*Head*. Width of head twice its median length; head dorsally smooth, strongly shiny and largely sparsely setose; antenna with 33 segments, 0.95× as long as forewing, third segment 1.6× as long as fourth segment, length of third, fourth and penultimate segments 2.6×, 1.7× and 1.6× their width, respectively; frons slightly depressed behind antennal sockets and smooth; eye in dorsal view 1.3× as long as temple; temple in dorsal view directly rounded, smooth and near eye sparsely setose; OOL: diameter of ocellus: POL= 3:1:2; face weakly convex, with distinctly longitudinal carina and downward pointing setae laterally, largely matt and punctulate; clypeus transverse, with sparse fine punctures and convex; eye glabrous; mandible nearly touching eye, length of malar space 0.1× basal width of mandible; mandible 1.4× as long as its maximum width, largely punctate-rugose medially; mandible weakly widened apically, with long and acute triangular middle tooth, and two wide lateral lobes (Fig. 46); maxillary palp 0.9× height of head.

*Mesosoma*. Length of mesosoma 1.5× its height; pronotum laterally mainly smooth except for sparse punctures and rugose posteriorly; propleuron without transverse carina subposteriorly, but slightly developed on left side; epicnemial area largely punctate; precoxal sulcus complete, moderately wide and distinctly crenulate; sternaulus absent; remainder of mesopleuron mostly smooth, dorsally punctate; postpectal carina medially not differentiated from mesosternal rugosity; episternal scrobe large; pleural sulcus finely crenulate dorsally and wider ventrally; mesosternal sulcus medium-sized and moderately crenulate, posteriorly rugose; metapleuron coarsely punctate-reticulate, but dorsally largely smooth; median groove of mesoscutum and notauli complete, narrow and finely crenulate, near posterior end punctate-crenulate; lateral lobes of mesoscutum mostly smooth, shiny, anterior half of middle lobe and area near notauli setose; scutellar sulcus deep and with 2 longitudinal carinae, 0.3× as long as scutellum; scutellum largely smooth, with few punctures and nearly flat; metanotal spine protruding far above level of scutellum; costulae and double median carina of propodeum distinctly developed but irregular, gradually lowered posteriorly and postero-laterally with protruding carinae; surface of propodeum partly smooth, medially with transverse band of coarse punctate-rugose.

*Wings*. Pterostigma elliptical; marginal cell of forewing distinctly elongate; vein r issued just before middle of pterostigma (Fig. 2); r:2-SR:3-SR+SR1 = 11:17:69; SR1 curved; 1-CU1:2-CU1 = 1:20; CU1b present; m-cu slightly antefurcal; 1-CU1 hardly widened. Hind wing: M+CU:1-M:1r-m = 28:13:12; cu-a straight; m-cu slightly impressed.

*Legs.* Hind coxa largely smooth and with long setae; tarsal claws medium-sized (Fig. 9); length of femur, tibia and basitarsus of hind leg 3.9, 9.1 and 5.0× their width, respectively; all femora widened.

*Metasoma*. Length of first tergite 1.2× its apical width, its surface longitudinally coarsely striate, with dorsal carinae converging medially and united in distinct median carina (Fig. 5); second tergite moderately striate, laterally finely striate; third metasomal tergite nearly entirely finely striate, moderately shiny, sparsely setose; combined length of second and third metasomal tergites 0.7× total length of metasoma (Fig. 6); striae of third tergite with distinct shiny interspaces.

*Colour.* Black (including mandible); palpi and legs yellow but tarsi and base of hind coxa darkened; tegulae, pterostigma and forewing veins dark brown; hind wing veins pale brown; forewing membrane slightly infuscate, hind wing nearly pellucid.

#### Variation.

Forewing length 3.2–3.3 mm, body length 3.3–3.4 mm; antenna with 33 (2 ♂) segments; subposterior transverse carina of propleuron absent or slightly developed; propodeum largely smooth anteriorly or distinctly punctate; paratype has sculpture of metasoma somewhat reduced medio-dorsally.

#### Distribution.

China (Shaanxi).

#### Etymology.

The name is derived from “a” (Greek for “not, without”) and “carina” (Latin for “ridge”) because of the lacking subposterior transverse carina of the propleuron.

### Trachionus albitibialis

Taxon classificationAnimaliaHymenopteraBraconidae

Cui & van Achterberg sp. n.

http://zoobank.org/56FD308B-D114-4836-91ED-0762A4009D1F

Figs 11 , 12–21 , 43

#### Type material.

Holotype, ♀ (NWUX), “NW. **China**: Shaanxi, Pingheliang, Ningshan, c. 2000 m, 27.vi.2011, 33°48'N, 108°50'E, Jiangli Tan, NWUX”.

#### Diagnosis.

Scutellum largely smooth, at most punctulate and nearly flat; mandible mainly brown, flat, without crest and with small fourth and fifth teeth (Figs 17, 43); temple smooth (Fig. 18); propleuron with transverse carina subposteriorly; sternaulus absent; median groove of mesoscutum and notauli rather narrow posteriorly (Fig. 14); metanotal spine medium-sized, its highest point remaining below level of tips of setae of scutellum (Fig. 21); propodeum angularly lowered posteriorly in lateral view and carina hardly protruding postero-laterally (Fig. 14); transverse carina of propodeum indistinct or absent (Fig. 14); medial third of hind tibia ivory; basal half of second metasomal tergite strongly rugose-striate, with about 30 striae and very shiny, rugae of third tergite with indistinct interspaces. Similar to *Trachionus hians*, but that species has precoxal sulcus posteriorly and posterior part of notauli wide (rather narrow in *Trachionus albitibialis*), basal half of hind tibia yellow (ivory) and basal half of second metasomal tergite largely finely aciculate (basal half of second tergite strongly rugose).

#### Description.

Holotype, female; body length 3.3 mm, forewing length 3.4 mm.

*Head*. Width of head twice its median length, head dorsally smooth, distinctly shiny and mostly sparsely setose; antenna with 31 (right) or 32 (left) segments, 1.1× longer than forewing, third segment 1.4× as long as fourth segment, length of third, fourth and penultimate segments 2.4×, 1.7× and 1.6× their width, respectively; frons with setae laterally and slightly depressed behind antennal sockets; eye in dorsal view 2.1× as long as temple; temple in dorsal view directly roundly narrowed, smooth and near mandible punctulate and punctate; OOL: diameter of ocellus: POL = 14:4:5; face weakly convex, punctulate, with many upward pointing setae medially and downward pointing setae laterally; clypeus transverse, with fine punctures and convex; eye glabrous; mandible nearly touching eye, length of malar space 0.1× basal width of mandible; mandible 1.3× as long as its maximum width and 1.6× as long as its basal width, mostly smooth, medially rugose; mandible with 4 teeth or lobes, and second one acute, wide triangular; maxillary palp 0.9× as long as height of head.

*Mesosoma*. Length of mesosoma 1.4× its height; pronotum laterally coarsely punctate, smooth dorsally and ventrally; propleuron with transverse carina subposteriorly; epicnemial area mostly coarsely punctate; precoxal sulcus complete, wide and distinctly punctate-crenulate; sternaulus absent; remainder of mesopleuron smooth and strongly shiny, but punctate dorsally; episternal scrobe large; pleural sulcus finely crenulate dorsally and coarser ventrally; mesosternal sulcus medium-sized and superficially crenulate, posteriorly punctate and with transverse rugae, but postpectal carina absent; metapleuron coarsely rugose-punctate; notauli rather narrow, completely punctate-crenulate and united medio-posteriorly; median groove of mesoscutum narrow posteriorly, punctate-crenulate; lateral lobes of mesoscutum smooth, shiny and glabrous, only anterior half of middle lobe and area near notauli setose; scutellar sulcus deep and with 4 longitudinal carinae, 0.4× as long as scutellum; scutellum finely punctate, with lateral setae and nearly flat; propodeum angularly lowered posteriorly in lateral view and carina hardly protruding postero-laterally (Fig. 14); transverse carina of propodeum indistinct or absent (Fig. 14); surface of propodeum largely rugose-reticulate.

*Wings*. Pterostigma elliptical; marginal cell of forewing elongate; vein r issued before middle of pterostigma (Fig. 12); r:2-SR:3-SR+SR1 = 6:11:46; SR1 curved; 1-CU1:2-CU1 = 2:19; CU1b present; m-cu antefurcal; 1-CU1 wide. Hind wing: M+CU:1-M:1r-m = 15:7:8; cu-a straight; m-cu only slightly impressed.

*Legs*. Hind coxa largely smooth and with long setae; tarsal claws medium-sized (Fig. 20); length of femur, tibia and basitarsus of hind leg 3.7, 7.8 and 4.6× their width, respectively; all femora widened.

*Metasoma*. Length of first tergite 0.8× its apical width, its surface punctate-rugose, with dorsal carinae converging medially and united in distinct median carina (Fig. 15); second tergite rather coarsely rugose-striate; basal half of third tergite coarsely rugose-striate and strongly shiny, rugae of third tergite with indistinct interspaces, apical part of third tergite smooth and shiny; combined length of second and third tergites 0.7× total length of metasoma (Fig. 16); setose part of ovipositor sheath 0.1× as long as fore wing; hypopygium large and apically acute (Fig. 16).

*Colour*. Black; mandible reddish brown; palpi pale yellow; basal two thirds of hind tibia ivory and apex infuscate, and tarsi largely dark brown; tegula brown; humeral plate and remainder of legs yellow; hypopygium black medially and remainder largely yellowish; pterostigma and most veins dark brown; wing membrane slightly infuscate, but hind wing nearly pellucid.

#### Distribution.

China (Shaanxi).

#### Etymology.

The name is derived from “albus” (Latin for “white”) and “tibia” (Latin for “shinbone”) because of the largely whitish hind tibia.

### Trachionus brevisulcatus

Taxon classificationAnimaliaHymenopteraBraconidae

Cui & van Achterberg sp. n.

http://zoobank.org/2F9F0FC6-C6A7-402F-B5D9-B7F16AF283AE

Figs 22 , 23–32 , 45

#### Type material.

Holotype, ♀ (NWUX), “NW. **China**: Shaanxi, Pingheliang, Ningshan, c. 2000 m, 27.vi.2011, 33°48'N, 108°50'E, Jiangli Tan, NWUX”. Paratypes (ZJUH, RMNH): 4 ♀, topotypic and same date.

#### Diagnosis.

Scutellum largely smooth, at most punctulate and nearly flat; mandible black, medially with irregular transverse crest and with minute fourth and fifth teeth (Figs 28, 30, 45); temple smooth and medium-sized (Fig. 29); propleuron without transverse carina subposteriorly; sternaulus absent; median groove of mesoscutum and notauli wide posteriorly; metanotal spine medium-sized, its highest point remaining below level of tips of setae of scutellum (Fig. 32); propodeum angularly lowered posteriorly in lateral view and carina hardly protruding postero-laterally (Fig. 25); transverse carina of propodeum coarsely developed and irregular; medial third of hind tibia brownish yellow; basal half of second metasomal tergite coarsely rugose-striate, with about 30 striae and very shiny (Fig. 26); rugae of third tergite with distinct shiny interspaces. Similar to *Trachionus hians*, but this species has a strong transverse carina at the propleuron subposteriorly (without transverse carina in *Trachionus brevisulcatus*) and basal half of second metasomal tergite largely finely aciculate (basal half of second tergite moderately striate).

#### Description.

Holotype, female; body length 3.5 mm, forewing length 3.5 mm.

*Head*. Width of head 2.1× its median length, head dorsally smooth, strongly shiny and largely sparsely setose; antenna with 37 segments,1.2× longer than fore wing, third segment 1.3× as long as fourth segment, length of third, fourth and penultimate segments 2.9×, 2.3× and 1.4× their width, respectively; frons slightly depressed behind antennal sockets and smooth; eye in dorsal view 2.2× as long as temple; temple in dorsal view directly roundly narrowed, smooth and near mandible punctulate; OOL: diameter of ocellus: POL= 10:3:3; face weakly convex, with long downward pointing setae laterally, punctulate and largely smooth; clypeus hemi-circular, smooth with some fine punctures and convex; eye glabrous; mandible nearly touching eye, length of malar space less than 0.1× basal width of mandible; mandible 1.1× as long as its maximum width and 1.2× as long as its basal width, largely rugose medially and basally; mandible with irregular transverse crest, two wide lateral lobes and minute ventral fourth and fifth teeth; maxillary palp as long as height of head.

*Mesosoma*. Length of mesosoma 1.5× its height; pronotum laterally punctate; propleuron without transverse carina subposteriorly; epicnemial area largely punctate; precoxal sulcus complete, wide and coarsely crenulate; sternaulus absent; remainder of mesopleuron smooth, but dorsally punctate; episternal scrobe large; pleural sulcus finely crenulate; mesosternal sulcus medium-sized and moderately crenulate, posteriorly widely reticulate; metapleuron narrowly smooth anteriorly and mainly coarsely punctate-reticulate; notauli deep and completely crenulate, united medio-posteriorly in wider reticulate area; median groove of mesoscutum complete and crenulate, mesoscutum smooth, shiny and only anterior half of middle lobe and area near notauli setose; scutellar sulcus very deep and with 3 longitudinal carinae, 0.4× as long as scutellum; scutellum largely smooth, with few fine punctures and nearly flat; metanotal spine medium-sized, its highest point remaining below level of tips of setae of scutellum (Fig. 32); propodeum angularly lowered posteriorly in lateral view and carina hardly protruding postero-laterally (Fig. 25); transverse carina of propodeum coarsely developed and irregular; surface of propodeum mostly reticulate and medio-longitudinal carina present anteriorly.

*Wings*. Pterostigma elliptical; marginal cell of forewing elongate; vein r issued after middle of pterostigma (Fig. 23); r:2-SR:3-SR+SR1 = 6:10:42; SR1 curved; 1-CU1:2-CU1 = 2:19; CU1b present; m-cu antefurcal; 1-CU1 hardly widened; M+CU1 sclerotized. Hind wing: M+CU:1-M:1r-m = 33:14:20; cu-a straight; m-cu vaguely indicated.

*Legs*. Hind coxa largely smooth and with long setae; tarsal claws medium-sized (Fig. 31); length of femur, tibia and basitarsus of hind leg 3.7, 6.0 and 3.9× their width, respectively; all femora widened.

*Metasoma*. Length of first tergite 1.1× its apical width, its surface punctate-rugose, with dorsal carinae converging medially and united in distinct median carina (Fig. 26); second tergite strongly rugose; basal half of third metasomal tergite moderately striate and strongly shiny, remainder smooth; combined length of second and third metasomal tergites 0.7× total length of metasoma (Figs 26–27); rugae of third metasomal tergite with distinct shiny interspaces; setose part of ovipositor sheath 0.1× as long as fore wing; hypopygium large and apically acute (Fig. 27).

*Colour*. Black (including mandible); palpi, tegulae and legs yellow, but tarsi and base of coxa darkened; hypopygium partly brown; pterostigma and veins dark brown; wing membrane slightly infuscate.

#### Variation.

Forewing length 3.5–3.6 mm, body length 3.5–3.9 mm; antenna 35 (1 ♀), 36 (1 ♀) and 37 (1 ♀) segments; hypopygium largely brownish yellow or dark brown.

#### Distribution.

China (Shaanxi).

#### Etymology.

The name is derived from “brevis” (Latin for “short”) and “sulcus” (Latin for “groove”) because of the short widened parts of precoxal sulcus and notauli.

### Trachionus mandibularoides

Taxon classificationAnimaliaHymenopteraBraconidae

Cui & van Achterberg sp. n.

http://zoobank.org/AF762251-A01A-476F-A32B-35D6FC76FBE8

Figs 33 , 34–42 , 44

#### Type material.

Holotype, ♀ (NWUX), “NW. **China**: Shaanxi, Xunyangba, Ningshan, c. 1300 m, vii.2014, 33°33'N, 108°32'E, Jiangli Tan, NWUX”.

#### Diagnosis.

Mandible with two wide lateral lobes and one smaller ventral lobe (Fig. 44); sternaulus present as flat punctate area anteriorly (Fig. 35); scutellum coarsely punctate and distinctly convex (Fig. 36); median punctate band of mesoscutum anteriorly as wide as smooth bands next to it (Fig. 36). The new species differs from the similar European *Trachionus mandibularis* (Nees, 1816) by having the epistomal suture narrow, the sternaulus not impressed (but indicated as a flat punctate area anteriorly) and the mandible distinctly widened ventrally.

#### Description.

Holotype, female; body length 2.2 mm, forewing length 2.7 mm.

*Head*. Width of head 2.5× its median length; dorsally head smooth, strongly shiny and only sparsely setose; antenna incomplete, 20 segments remaining, third segment 1.6× as long as fourth segment, length of third and fourth segments 2.3× and 1.5× their width, respectively; frons narrowly depressed behind antennal sockets and smooth; eye in dorsal view 2.2× longer than temple; temple in dorsal view rounded, shiny and sparsely setose; OOL: diameter of ocellus: POL = 10:3:4; face medio-dorsally somewhat elevated, with upward pointing long setae, with satin sheen and punctulate; clypeus transverse, with some small punctures and convex; epistomal suture narrow; mandible nearly touching eye, length of malar space 0.1× basal width of mandible; mandible 1.3× as long as its maximum width, largely rugose medially; mandible distinctly widened apically, with long and acute triangular middle tooth, two wide lateral lobes and one smaller lobe ventrally; maxillary palp 0.9× height of head.

*Mesosoma*. Length of mesosoma 1.4× its height; pronotum laterally mostly punctate, but smooth medio-dorsally; sternaulus present as spaced punctate area anteriorly; precoxal sulcus complete, wide and rugose-punctate; remainder of mesopleuron largely smooth but punctate dorsally; mesosternal sulcus shallow and punctate, posteriorly transversely rugose; metapleuron coarsely punctate-reticulate; median groove of mesoscutum and notauli complete, wide and distinctly punctate, mesoscutum anteriorly rugose-punctate; lateral lobes of mesoscutum glabrous, smooth and shiny medially; remainder of mesoscutum with few setae; scutellar sulcus deep, punctate and with 3 longitudinal carinae, 0.4× as long as scutellum; scutellum convex, coarsely punctate and with long setae; highest point of metanotal spine protruding above level of scutellum; surface of propodeum coarsely foveolate-punctate, median carina of propodeum only anteriorly present, remainder of carinae indiscernible because of surrounding sculpture; propodeum rather steeply lowered posteriorly and postero-laterally with protruding carinae.

*Wings*. Pterostigma nearly elliptical; marginal cell of forewing elongate; vein r issued just before middle of pterostigma (Fig. 34); r:2-SR:3-SR+SR1 = 6:11:45; SR1 curved; 1-CU1:2-CU1 = 1:20; M+CU1:1-M:m-cu = 7:4:3; CU1b present; m-cu slightly antefurcal; 1-CU1 widened. Hind wing: M+CU:1-M:1r-m = 14:8:9; cu-a straight; m-cu absent.

*Legs*. Hind coxa mostly smooth; tarsal claws medium-sized (Fig. 41); length of femur, tibia and basitarsus of hind leg 3.2, 6.2 and 3.5× their width, respectively; all femora slightly widened.

*Metasoma*. Length of first tergite nearly equal to its apical width, its surface longitudinally coarsely striate, with dorsal carinae converging medially and united in distinct median carina (Fig. 37); second and third tergites punctate-striate, but third tergite smooth apically; combined length of second and third metasomal tergites 0.4× total length of metasoma (Figs 37, 42); apically striae of third tergite with distinct shiny interspaces; setose part of ovipositor sheath 0.1× as long as fore wing; hypopygium large and apically acute (Fig. 42).

*Colour*. Black (including mandible); palpi and legs yellow but tarsi dark brown and base of hind coxa darkened; tegulae, ovipositor sheath, pterostigma and forewing veins dark brown; veins of hind wing pale brown; forewing membrane slightly infuscate; metasoma dark brown ventrally, but hypopygium yellowish brown apico-laterally.

#### Distribution.

China (Shaanxi).

#### Etymology.

The new species is named after *Trachionus mandibularis* and “oides” (Latin for “similar to”), because of the similar sculpture of the mesosoma.

## Supplementary Material

XML Treatment for Trachionus

XML Treatment for Trachionus acarinatus

XML Treatment for Trachionus albitibialis

XML Treatment for Trachionus brevisulcatus

XML Treatment for Trachionus mandibularoides
